# Do antihypertensive drugs really have antitumor effects? Baseline differences in hypertensive and non-hypertensive patients with advanced pancreatic cancer

**DOI:** 10.1097/MD.0000000000030451

**Published:** 2022-08-26

In the article, “Do antihypertensive drugs really have antitumor effects? Baseline differences in hypertensive and non-hypertensive patients with advanced pancreatic cancer”,^[1]^ which appears in Volume 101, Issue 29 of *Medicine*, the bold p values were not maintained in the final version of the paper in Tables 3 and 4. This has since been corrected.
